# Differences in carbon and nitrogen metabolism of soft japonica rice in southern China during grain filling stage under different light and nitrogen fertilizer conditions and their relationship with rice eating quality

**DOI:** 10.3389/fpls.2025.1534625

**Published:** 2025-01-28

**Authors:** Zhongtao Ma, Jiale Cao, Xi Chen, Jianghui Yu, Liu Guodong, Fangfu Xu, Qun Hu, Guangyan Li, Ying Zhu, Hongcheng Zhang, Haiyan Wei

**Affiliations:** ^1^ Jiangsu Key Laboratory of Crop Genetics and Physiology, Agricultural College of Yangzhou University, Yangzhou, China; ^2^ Jiangsu Key Laboratory of Crop Cultivation and Physiology, Agricultural College of Yangzhou University, Yangzhou, China; ^3^ Jiangsu Co-Innovation Center for Modern Production Technology of Grain Crops, Yangzhou University, Yangzhou, China; ^4^ Research Institute of Rice Industrial Engineering Technology of Yangzhou University, Yangzhou, China

**Keywords:** light intensity, nitrogen fertilizer, carbon metabolism nitrogen metabolism, C/N, eating quality, soft japonica rice

## Abstract

Light and nitrogen are crucial environmental factors that significantly impact rice growth and quality formation. Currently, there is a lack of systematic research on how light and nitrogen affect carbon and nitrogen metabolism during grain filling, subsequently affecting the eating quality of rice. To address this gap, field experiments were conducted under varying light intensities and nitrogen fertilizer levels to investigate the changes in carbon and nitrogen metabolism during grain filling, the eating quality of rice at maturity, and the relationship between them. The findings revealed that, 50% light intensity suppressed carbon metabolism while stimulating nitrogen metabolism, resulting in a reduction in the C/N ratio, decreased starch content by 4.30% to 5.59%, and elevated protein content by 21.31% to 29.70%, thereby leading to decreased rice eating quality by 10.06% to 11.42%. Conversely, the application of panicle fertilizer boosted nitrogen metabolism while hindering carbon metabolism, leading to a decrease in the C/N ratio, increased protein content by 21.31% to 29.70%, and reduced starch content by1.60% to 2.93%, thereby leading to decreased rice eating quality by 4.13% to 6.71%. Correlation analysis revealed a significant positive correlation between the C/N ratio and carbon metabolism-related enzyme activities and products, along with a significant negative correlation with nitrogen metabolism-related enzyme activities and products, suggesting that the C/N ratio can serve as an indicator of carbon and nitrogen metabolism levels. Further analysis revealed a significant positive relationship between the C/N ratio and taste value, indicating that higher levels of carbon metabolism promote the development of good rice eating quality, while nitrogen metabolism exerts an opposing influence. In summary, notable variances in carbon and nitrogen metabolism were observed within the same japonica rice cultivar under diverse light and nitrogen fertilizer conditions. These metabolic differences impact the synthesis of starch and protein in the endosperm, ultimately influencing rice quality. Our study contributes to a more profound comprehension of the regulation of carbon and nitrogen metabolism in rice by light and nitrogen fertilizer, as well as their role in determining eating quality.

## 1 Introduction

China is recognized as the world’s largest producer of japonica rice, with the southern rice region serving as the primary cultivation area for japonica rice production in the country. This region is renowned for its high yield per unit area and substantial total output, playing a crucial role in ensuring a stable national food supply (Zhu et al., 2021). Conventional japonica rice varieties grown in the southern regions have historically exhibited poor rice eating quality, characterized by high hardness and low viscosity. However, recent years have witnessed significant advancements in breeding new soft japonica rice varieties with low amylose content in the southern japonica rice regions (Ma et al., 2022; Zhu et al., 2021). In contrast to conventional japonica rice, these new soft japonica rice varieties demonstrate a softer and more elastic texture after cooking, thereby significantly enhancing the eating quality of southern japonica rice (Zhu et al., 2022).

Starch and protein are key determinants of rice eating quality (Li and Gilbert, 2018; Scott and Awika, 2023; Yu et al., 2023), with their synthesis and accumulation being regulated by carbon and nitrogen metabolism during the grain filling stage (Pfister and Zeeman, 2016; Zhang and Fernie, 2023). Thus, modifications in carbon and nitrogen metabolism are critical for the content and composition of starch and protein in rice endosperm, thereby influencing the overall rice eating quality. Previous research has indicated that, beyond rice genotypes, environmental factors such as light and nitrogen fertilizer significantly impact carbon and nitrogen metabolism in rice (Li et al., 2022a; Wei et al., 2018). Insufficient light levels during the grain filling stage result in reduced activity of ribulose 1,5-bisphosphate carboxylase in the leaves, leading to a decline in leaf photosynthetic rate and insufficient accumulation of photosynthetic products (Carmo-Silva and Salvucci, 2013; Wei et al., 2023). Concurrently, the activities of starch synthesis enzymes in grains, including ADP-glucose pyrophosphorylase, granule-bound starch synthase, and soluble starch synthase, decrease in response to diminishing light intensity (Deng et al., 2018; Li et al., 2022b). This decline results in reduced starch content in the grains, coupled with an increase in long-chain amylopectin (Deng et al., 2021). Moreover, certain investigations have indicated that inadequate light exposure can augment nitrogen accumulation in rice plants, facilitating protein synthesis in grains and consequently elevating the protein content. Nitrogen stands as a vital nutrient essential for rice cultivation. Proper nitrogen fertilizer application has been demonstrated to delay leaf senescence, enhance leaf photosynthetic efficiency, and improve the assimilation and uptake of carbon and nitrogen compounds in plants (Tanaka et al., 2022; Zhang et al., 2021a). A previous investigation revealed that the delayed application of nitrogen fertilizer enhances nitrogen absorption by rice plants and augments the leaf net photosynthetic rate. This phenomenon not only boosts nitrogen and non-structural carbohydrate (NSC) accumulation in the stems post-anthesis but also enhances their efficacy in transporting these compounds to the grains. Such processes furnish an ample supply of materials for starch and protein synthesis in the endosperm (Sun et al., 2017). Nevertheless, heightened nitrogen fertilizer application frequently elevates the activities of enzymes involved in protein synthesis in grains, such as glutamine synthetase and glutamate oxaloacetate transaminase, while concurrently suppressing the activities of enzymes associated with starch synthesis. Consequently, this leads to an imbalance in the starch-to-protein ratio within the rice endosperm (Fei et al., 2023; Long et al., 2023).

While previous studies have investigated carbon and nitrogen metabolism during the filling stage and its impact on rice eating quality, most have concentrated on the impact of light and nitrogen fertilizer on carbon and nitrogen metabolism in traditional rice varieties. Additionally, existing research has predominantly examined the response of rice eating quality to individual factors such as carbon or nitrogen metabolism. However, there remains a notable gap in comprehensive research exploring how light and nitrogen fertilizer modulate carbon and nitrogen metabolism in japonica rice during the grain filling stage, particularly in southern soft japonica rice. Furthermore, there is a lack of comprehension regarding how these factors influence the synthesis of starch and protein in the endosperm and the resultant eating quality. To address this research gap, the present study utilized two widely cultivated soft japonica rice cultivars in southern China, namely Nanjing 5718 and Nanjing 9108, as experimental materials and exposed them to different light and nitrogen fertilizer conditions. This investigation explores multiple parameters, including leaf photosynthetic rate, NSC and nitrogen content in stems and grains, starch and protein content in grains, activities of enzymes associated with starch and protein synthesis, and the eating quality of rice across varied light and nitrogen fertilizer conditions. The main objective is to thoroughly elucidate the mechanisms through which light and nitrogen fertilizer modulate carbon and nitrogen metabolism during the grain filling stage of southern soft japonica rice, consequently influencing the synthesis of starch and protein and the eventual eating quality of rice.

## 2 Materials and methods

### 2.1 Materials and experimental design

The experiment was conducted from May to November of 2021 at the experimental farm of Yangzhou University. Two widely cultivated soft japonica rice cultivars in southern China, Nanjing 5718 and Nanjing 9108, were selected as the experimental materials. A split-plot design was implemented, with cultivars serving as the main plots and light intensity and nitrogen fertilizer levels as the subplots. During the grain filling stage, two light intensity conditions were established: 100% natural light intensity (L1) and 50% natural light intensity (L2, achieved by shading with black shade nets with a transmittance of 50% from rice heading to maturity). The black shading net was customized by us from a supplier. Additionally, we conducted measurements using a lux meter to confirm its effectiveness in providing 50% shading. In conventional agricultural practices for high-quality and high-yield production in southern China, the application of 270 kg/hm² of pure nitrogen was employed, with a base fertilizer: tillering fertilizer: panicle fertilizer ratio of 3.5:3.5:3. Concerning the nitrogen application rate specified above, two panicle fertilizer levels were established: N1 (189 Kg N/hm² with 94.5 Kg each for base and tillering fertilizer, and no panicle fertilizer) and N2 (270 Kg N/hm² with 94.5 Kg each for base and tillering fertilizer, and 81 Kg for panicle fertilizer). Mechanical rice planting utilized blanket seedlings, with each hill accommodating four seedlings positioned at 30 cm by 12 cm intervals. The tillering fertilizer was administered 7 days post-transplantation, while the panicle fertilizer was applied at the fourth leaf stage from the top for each cultivar. The nitrogen fertilizer (N): phosphorus fertilizer (P_2_O_5_): potassium fertilizer (K_2_O) ratio was maintained at 2:1:2. Phosphorus fertilizer was applied as a single base application, whereas potassium fertilizer was evenly distributed before tillage and at the jointing stage.

### 2.2 Measurement of photosynthetic characteristics and SPAD

From anthesis to 42 days post-anthesis, designated rice plants that had reached flowering on the corresponding day were selected every 7 days. The net photosynthetic rate of the flag leaf was assessed utilizing a LI-6400XT portable photosynthesis system following the methodology outlined by Ye et al. (2024). Consistent positioning on the flag leaf was maintained for measurements across all treatments. The SPAD values of the flag leaf were determined using a SPAD-502 chlorophyll meter (Konica Minolta, Japan), with the average value being recorded.

### 2.3 Measurement of non-structural carbohydrates and nitrogen

Rice plants that flower simultaneously were marked, and the flowering period was recorded. Rice plant samples were performed at 7-day intervals from anthesis to 42 days post-anthesis. Subsequently, the rice plant was divided into stem, leaf and panicle. Enzyme inactivation was carried out at 105°C for 0.5 hours, followed by drying at 80°C until a consistent weight was attained. After drying and weighing, the samples were ground for the analysis of starch, soluble sugars, and nitrogen content.

Soluble sugars and starch levels were measured following the methodology outlined by Shouichi Yoshida et al. (1976). A 0.1 g sample was weighed into a 10 mL centrifuge tube, and 8 mL of a prepared 80% ethanol solution was added. The mixture underwent a 30-minute incubation in a water bath at 80°C, followed by cooling and centrifugation. This process was repeated thrice, and the supernatants were pooled for the determination of soluble sugars. The residue was utilized for starch determination. The anthrone colorimetric method was applied at 620 nm to analyze the content of soluble sugars and starch. Nitrogen content was determined using an automatic Kjeltec 8200 instrument (Foss, Hillerød, Denmark).

### 2.4 Measurement of carbon and nitrogen metabolism enzymes

Rice plants that flower simultaneously were marked, and the flowering period was recorded. Leaf and grain samples were performed at 7-day intervals from anthesis to 42 days post-anthesis. The leaf and grain samples were promptly frozen and preserved at -70°C for subsequent enzyme analysis.

Upon grain shelling, the activities of ADP-glucose pyrophosphorylase (AGPase), granule-bound starch synthase (GBSS), soluble starch synthase (SSS), starch branching enzyme (SBE), starch debranching enzyme (DBE), glutamine synthetase (GS), and glutamate synthase (GOGAT) were individually assessed. Subsequently, upon the main vein removal from the flag leaf, the activities of ribulose 1,5-bisphosphate carboxylase (RuBPase) and nitrate reductase (NR) were determined. All enzyme activities were measured in accordance with the protocols outlined in the respective kits provided by Shanghai Enzyme-linked Biotechnology Co., Ltd.

### 2.5 Measurement of starch, protein, and their components

Rice plants that flower simultaneously were marked, and the flowering period was recorded. Grain samples were performed at 7-day intervals from anthesis to 42 days post-anthesis. The grain samples were air-dried to achieve a moisture content of 14%, after which the quantities of starch, amylose, amylopectin, protein, albumin, globulin, prolamin, and glutelin were assessed.

The total starch content (TSC) was measured utilizing the Total Starch Assay Kit from Megazyme (Bray, Ireland). Amylose content (AC) was determined through the iodine adsorption method. The amylopectin content (APC) was calculated as the difference between TSC and AC. The crude protein content (PC) was analyzed using the Kjeldahl method with a Kjeltec 8200 analyzer (Foss, Hillerød, Denmark). Protein components were evaluated following the procedure outlined by Sapan et al. (1999), with glutelin being determined using a biuret colorimetric method, and albumin, globulin, and prolamin being quantified utilizing the Coomassie Blue colorimetric method (Ma et al., 2024).

### 2.6 Accumulation of starch, protein, and their components

The accumulation of starch and protein was modeled using the logarithmic equation y=K/(1+e^a−bx^) (Huang et al., 2019). Subsequently, the pertinent indicators were computed following the methodology outlined by Shi et al. (2012). The mean accumulation rate (MAR) was determined as Kb/(a-log_e_ (100/99-1)).

### 2.7 Rice eating quality

The cooked rice taste parameters, encompassing appearance and taste value, were evaluated utilizing a rice taste analyzer (STA1A, Satake Corporation, Japan), with the preset detection line configured as “Japanese japonica rice” (Zhu et al., 2021). The rice-to-water ratio was standardized at 1:1.3. Three rice cakes per sample were analyzed, with each side being assessed once.

The hardness, elasticity, and adhesiveness of cooked rice were assessed employing a texture analyzer (TA.XT.Plus, Stable Micro Systems, UK). The probe underwent a controlled descent and retraction at a velocity of 1 mm/s, followed by a repetitive compression cycle (Liu et al., 2021). Each sample underwent six analyses, and the resulting average values were calculated.

### 2.8 Data analysis

Data processing was carried out using EXCEL 2019, and statistical analysis was performed using SPSS 20.0 software. Graphing and correlation analyses were conducted using Origin 2022 software.

## 3 Results

### 3.1 Differences in carbon metabolism and its products under different light and nitrogen fertilizer conditions

#### 3.1.1 Photosynthetic characteristics of flag leaf

**Figure 1:**
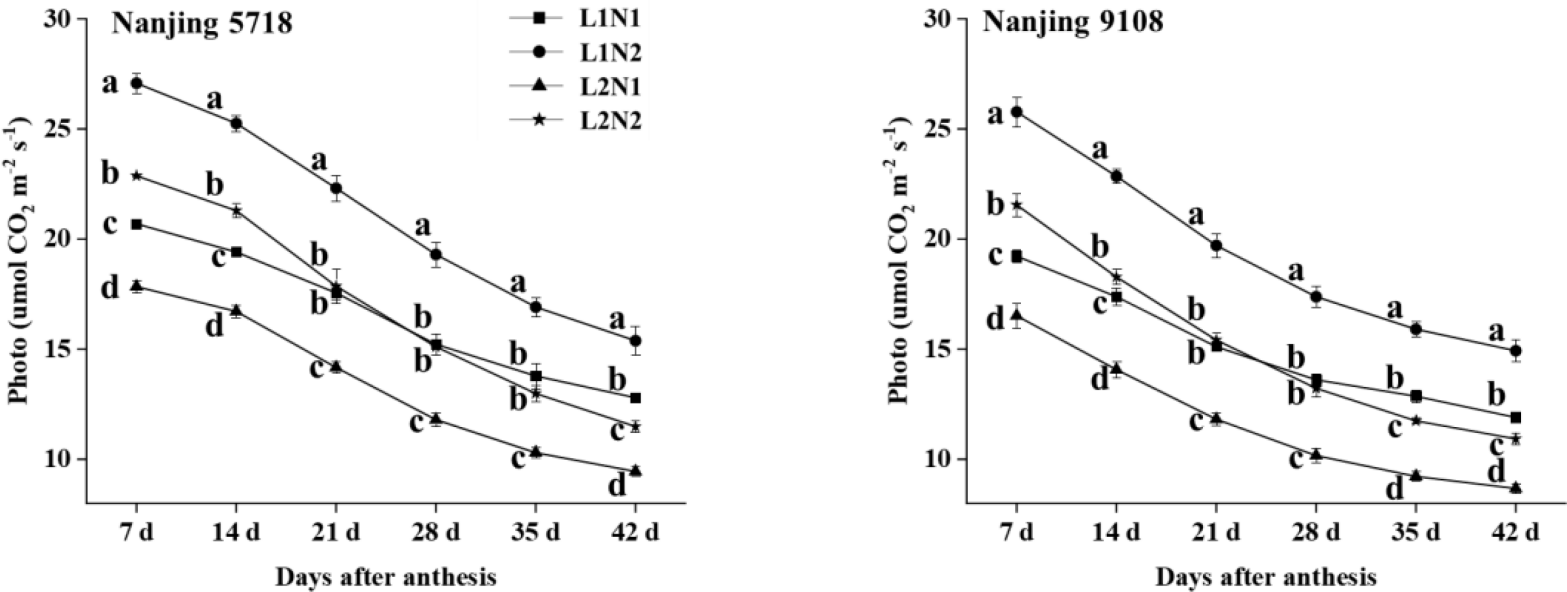
Dynamics of photo in the flag leaf of japonica rice. Values are mean ± SD (n = 3) and values in the same column with different letters are significantly different (*p* < 0.05). 100% natural light intensity without panicle fertilizer (L1N1), 100% natural light intensity with panicle fertilizer (L1N2), 50% natural light intensity without panicle fertilizer (L2N1) and 50% natural light intensity with panicle fertilizer (L2N2).

Under equivalent nitrogen conditions, the photosynthesis rate of L2 decreased by 13.68% to 16.28%, 13.90% to 16.40%, 19.06% to 20.01%, 21.70% to 22.51%, 23.23% to 25.30%, 25.32% to 28.30%, and 25.80% to 27.10%, respectively, from 7 days to 42 days after anthesis (**Figure 1**).

Under consistent light conditions, the photosynthesis rate of N2 compared to N1 exhibited an increase ranging from 28.20% to 35.39%, 27.39% to 34.11%, 26.02% to 31.50%, 26.79% to 30.49%, 22.71% to 30.07%, 20.24% to 27.37%, and 20.21% to 26.00%, respectively, from 7 days to 42 days after anthesis.

#### 3.1.2 The content and accumulation of non-structural carbohydrates in stems

Under equivalent nitrogen conditions, the non-structural carbohydrate (NSC) content and accumulation of L2 at the maturity stage decreased by 8.76% to 15.37% and 17.51% to 19.74%, respectively, compared to L1 (**Table 1**).

**Table 1:** Non-structural carbohydrate content and accumulation in the stems of japonica rice.

Cultivar	Treatment	Content (%)		Accumulation (Kg/hm^2^)	
		Anthesis stage	Maturity stage	Anthesis stage	Maturity stage
Nanjing5718	L1N1	33.60 ± 0.22a	19.51 ± 0.33a	1950.14 ± 12.80d	984.92 ± 16.67b
	L1N2	31.51 ± 0.14c	17.74 ± 0.23b	2023.74 ± 9.13b	997.14 ± 12.75b
	L2N1	33.31 ± 0.26a	17.16 ± 0.52b	1914.29 ± 15.22e	790.48 ± 23.79e
	L2N2	31.21 ± 0.39c	15.01 ± 0.28d	1986.01 ± 24.71c	810.05 ± 15.16de
Nanjing9108	L1N1	32.71 ± 0.18b	17.61 ± 0.34b	1967.29 ± 11.08cd	1012.83 ± 19.78ab
	L1N2	31.06 ± 0.52c	16.34 ± 0.27c	2064.06 ± 34.77a	1040.13 ± 16.97a
	L2N1	32.72 ± 0.27b	16.07 ± 0.49c	1938.79 ± 15.57de	835.47 ± 25.73cd
	L2N2	29.76 ± 0.32d	14.42 ± 0.18e	1989.00 ± 21.60c	850.98 ± 10.73c

Values are mean ± SD (n = 3) and values in the same column with different letters are significantly different (*p* < 0.05).

100% natural light intensity without panicle fertilizer (L1N1), 100% natural light intensity with panicle fertilizer (L1N2), 50% natural light intensity without panicle fertilizer (L2N1) and 50% natural light intensity with panicle fertilizer (L2N2).

Under consistent light conditions, the NSC content of N2 at maturity stage exhibited a decrease ranging from 7.22% to 12.53% compared to N1, while the accumulation of N2 at maturity stage showed an increase of 1.27% to 2.70%.

#### 3.1.3 The content of non-structural carbohydrates in grains

**Figure 2:**
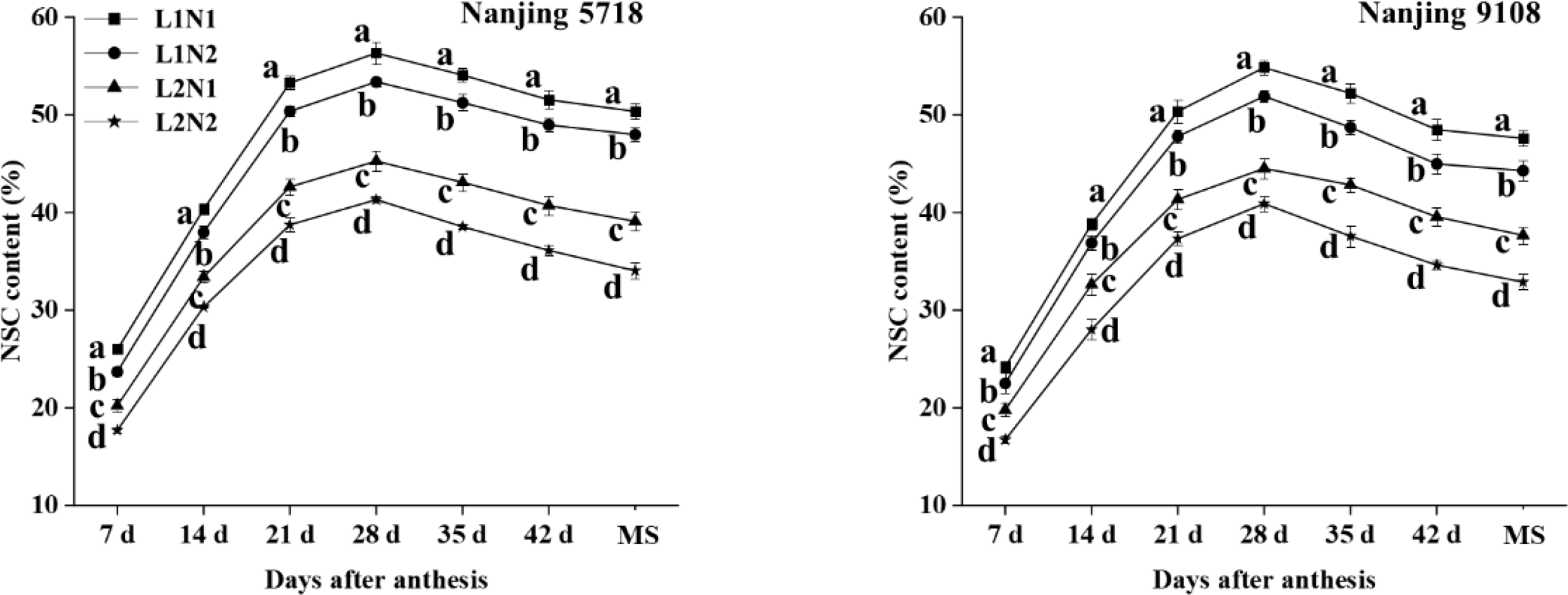
Dynamics of non-structural carbohydrate content in the grains of japonica rice. Values are mean ± SD (n = 3) and values in the same column with different letters are significantly different (*p* < 0.05). Non-structural carbohydrate (NSC) and maturity stage (MS). 100% natural light intensity without panicle fertilizer (L1N1), 100% natural light intensity with panicle fertilizer (L1N2), 50% natural light intensity without panicle fertilizer (L2N1) and 50% natural light intensity with panicle fertilizer (L2N2).

Under equivalent nitrogen conditions, the non-structural carbohydrate (NSC) content of L2 decreased by 18.17% to 25.69%, 15.95% to 24.03%, 17.88% to 23.09%, 18.84% to 22.60%, 17.97% to 24.79%, 18.41% to 26.22%, and 20.87% to 29.04%, respectively, from 7 days after anthesis to the maturity stage, in comparison to L1 (**Figure 2**).

Under consistent light conditions, in comparison to N1, the NSC content of N2 exhibited a decrease ranging from 6.88% to 15.44%, 4.96% to 14.10%, 5.09% to 9.75%, 5.21% to 8.70%, 5.22% to 12.28%, 5.00% to 12.46%, and 4.72% to 12.90%, respectively, from 7 days after anthesis to maturity stage.

#### 3.1.4 Enzyme activities of carbon metabolism in leaves and grains

**Figure 3:**
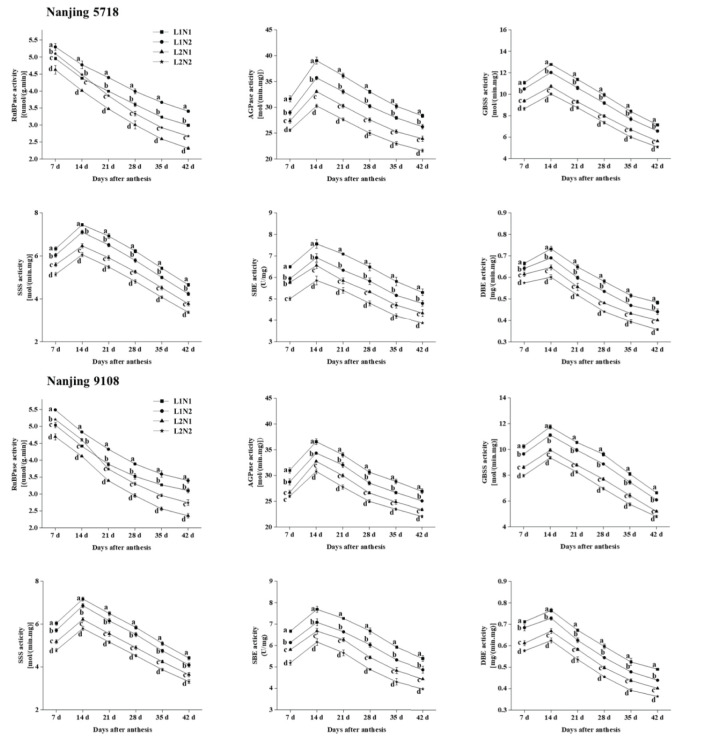
Dynamics of enzyme activities in the leaves and grains of japonica rice. Values are mean ± SD (n = 3) and values in the same column with different letters are significantly different (*p* < 0.05). 1,5-bisphosphate carboxylase (RuBPase), ADP-glucose pyrophosphorylase (AGPase), granule-bound starch synthase (GBSS), soluble starch synthase (SSS), starch branching enzyme (SBE) and starch debranching enzyme (DBE). 100% natural light intensity without panicle fertilizer (L1N1), 100% natural light intensity with panicle fertilizer (L1N2), 50% natural light intensity without panicle fertilizer (L2N1) and 50% natural light intensity with panicle fertilizer (L2N2).

Under equivalent nitrogen conditions, compared to L1, the levels of RuBPase in L2 leaves exhibited a decrease of 3.77% to 24.03%, while the activities of AGPase, GBSS, SSS, SBE, and DBE in L2 grains decreased by 9.74% to 19.58%, 15.25% to 23.03%, 13.16% to 20.26%, 11.22% to 19.25%, and 7.63% to 18.90%, respectively, from 7 days to 42 days after anthesis (**Figure 3**).

Under consistent light conditions, in comparison to N1, RuBPase activity in N2 leaves increased by 6.87% to 16.39%, while the activities of AGPase, GBSS, SSS, SBE, and DBE in N2 grains decreased by 2.76% to 10.19%, 5.16% to 10.63%, 4.37% to 10.45%, 7.61% to 13.18%, and 3.53% to 10.89%, respectively, from 7 days to 42 days after anthesis.

#### 3.1.5 Characterization of starch content and accumulation in brown rice

**Figure 4:**
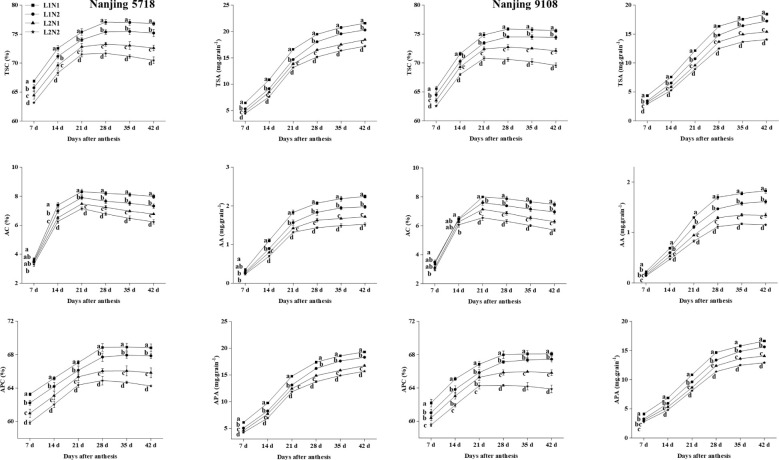
Dynamics of starch in the grains of japonica rice. Values are mean ± SD (n = 3) and values in the same column with different letters are significantly different (*p* < 0.05). Total starch content (TSC), total starch accumulation (TSA), amylose content (AC), amylose accumulation (AA), amylopectin content (APC) and amylopectin accumulation (APA). 100% natural light intensity without panicle fertilizer (L1N1), 100% natural light intensity with panicle fertilizer (L1N2), 50% natural light intensity without panicle fertilizer (L2N1) and 50% natural light intensity with panicle fertilizer (L2N2).

Under equivalent nitrogen conditions, in comparison to L1, the TSC, AC and APC in L2 decreased by 2.82% to 6.25%, 3.62% to 18.06%, and 1.86% to 5.44%, respectively, from 7 days to 42 days after anthesis (**Figure 4**). Additionally, due to the reduced MAR of starch, amylose, and amylopectin in L2 compared to L1 (**Table 2**), the TSA, AA, and APA of L2 decreased by 14.19% to 18.59%, 23.01% to 28.56%, and 13.46% to 17.57%, respectively, at 42 days after anthesis (**Figure 4**).

**Table 2:** Characteristics of starch accumulation.

Cultivar	Treatment	Total starch			Amylose			Amylopectin		
		MAR (mg grain^-1^ d^-1^)	K	R^2^	MAR (mg grain^-1^ d^-1^)	K	R^2^	MAR (mg grain^-1^ d^-1^)	K	R^2^
Nanjing5718	L1N1	0.4775	21.92	0.99616	0.0646	2.20	0.99887	0.4141	19.76	0.99500
	L1N2	0.4369	20.81	0.99681	0.0550	1.94	0.99887	0.3828	18.90	0.99576
	L2N1	0.4123	18.62	0.99454	0.0503	1.71	0.99932	0.3626	16.97	0.99394
	L2N2	0.3888	17.31	0.98803	0.0445	1.51	0.99784	0.3432	15.86	0.98745
Nanjing9108	L1N1	0.3828	19.13	0.99440	0.0472	1.83	0.99918	0.3359	17.35	0.99331
	L1N2	0.3549	17.93	0.99741	0.0402	1.62	0.99945	0.3149	16.35	0.99671
	L2N1	0.3251	16.11	0.99224	0.0348	1.36	0.99721	0.2901	14.79	0.99115
	L2N2	0.3011	14.64	0.99339	0.0293	1.15	0.99780	0.2706	13.50	0.99236

Starch accumulation was modeled using the logarithmic equation y=K/(1+ea−bx). K, the max accumulation. R2, determination coefficient of the equation. Mean accumulation rate (MAR). 100% natural light intensity without panicle fertilizer (L1N1), 100% natural light intensity with panicle fertilizer (L1N2), 50% natural light intensity without panicle fertilizer (L2N1) and 50% natural light intensity with panicle fertilizer (L2N2).

Under consistent light conditions, in comparison to N1, the TSC, AC, and APC in N2 decreased by 1.28% to 3.50%, 0.96% to 9.31%, and 0.68% to 2.95%, respectively, from 7 days to 42 days after anthesis. Similarly, reflecting the lower MAR of starch, amylose, and amylopectin in N2 compared to N1, the TSA, AA, and APA of N2 decreased by 5.94% to 8.73%, 11.54% to 14.23%, and 6.24% to 8.20%, respectively, at 42 days after anthesis.

### 3.2 Differences in nitrogen metabolism and its products under different light and nitrogen fertilizer conditions

#### 3.2.1 SPAD of flag leaf

**Figure 5:**
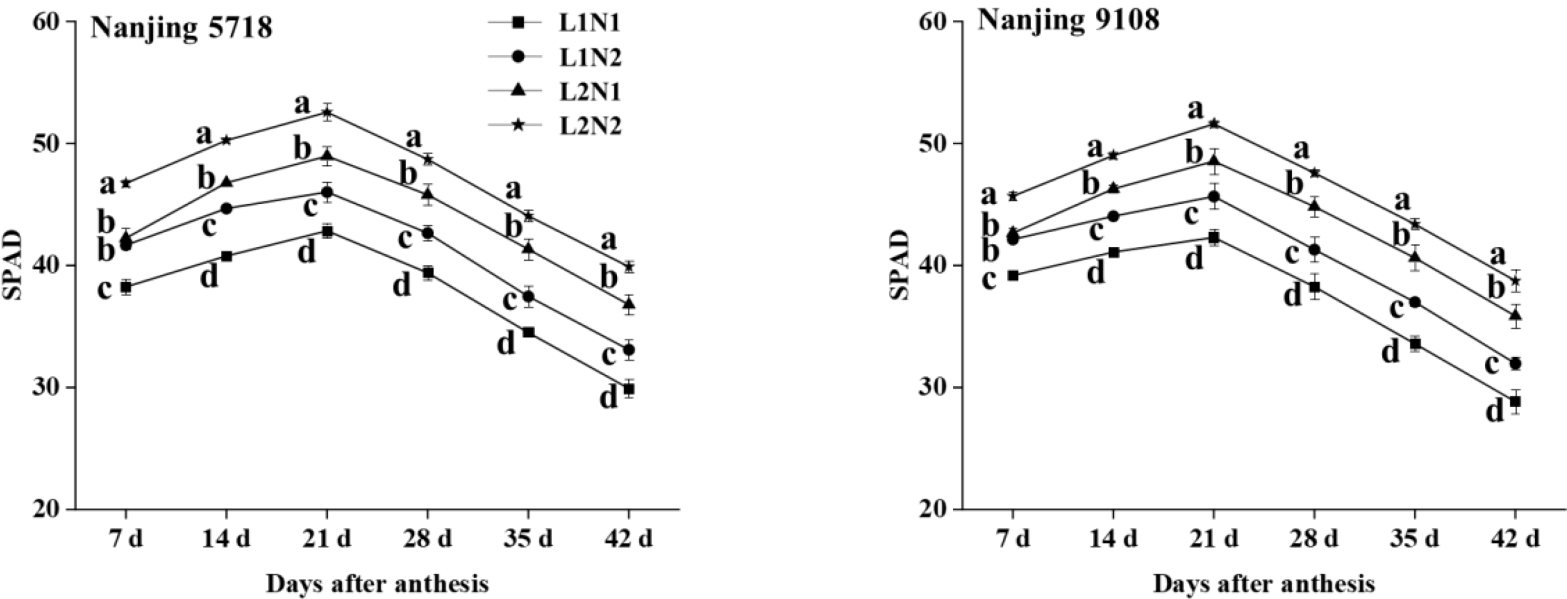
Dynamics of SPAD in the flag leaf of japonica rice. Values are mean ± SD (n = 3) and values in the same column with different letters are significantly different (*p* < 0.05). 100% natural light intensity without panicle fertilizer (L1N1), 100% natural light intensity with panicle fertilizer (L1N2), 50% natural light intensity without panicle fertilizer (L2N1) and 50% natural light intensity with panicle fertilizer (L2N2).

Under equivalent nitrogen conditions, compared to L1, SPAD of L2 increased by 8.40% to 12.19%, 11.31% to 14.71%, 13.01% to 14.75%, 14.16% to 17.15%, 17.24% to 20.96%, 20.58% to 24.34%, and 27.19% to 34.07%, respectively, from 7 days to 42 days after anthesis (**Figure 5**).

Under consistent light conditions, compared to N1, SPAD of N2 increased by 7.08% to 10.65%, 5.92% to 9.56%, 6.30% to 7.94%, 6.16% to 8.27%, 6.58% to 10.18%, 8.03% to 10.82%, and 7.48% to 13.30%, respectively, from 7 days to 42 days after anthesis.

#### 3.2.2 The content of nitrogen content and accumulation in stems

Under equivalent nitrogen conditions, the nitrogen content and accumulation of L2 at maturity stage increased by 15.88% to 20.00% and 8.50% to 12.71%, respectively, compared to L1 (**Table 3**).

**Table 3:** Nitrogen content and accumulation in the stems of japonica rice.

Cultivar	Treatment	Content (%)		Accumulation (Kg/hm^2^)	
		Anthesis stage	Maturity stage	Anthesis stage	Maturity stage
Nanjing5718	L1N1	0.75 ± 0.01c	0.47 ± 0.001f	43.36 ± 0.27d	23.87 ± 0.06h
	L1N2	0.87 ± 0.01b	0.57 ± 0.003d	56.04 ± 0.31b	32.21 ± 0.18d
	L2N1	0.75 ± 0.01c	0.56 ± 0.01d	42.83 ± 0.33d	25.98 ± 0.51g
	L2N2	0.88 ± 0.002b	0.66 ± 0.003b	56.14 ± 0.13b	35.82 ± 0.18c
Nanjing9108	L1N1	0.86 ± 0.004b	0.49 ± 0.01e	51.80 ± 0.23c	28.33 ± 0.44f
	L1N2	0.98 ± 0.03a	0.59 ± 0.01c	65.03 ± 1.72a	37.57 ± 0.85b
	L2N1	0.86 ± 0.004b	0.59 ± 0.01c	50.84 ± 0.25c	30.74 ± 0.52e
	L2N2	0.99 ± 0.01a	0.71 ± 0.01a	66.18 ± 0.59a	42.34 ± 0.53a

Values are mean ± SD (n = 3) and values in the same column with different letters are significantly different (*p* < 0.05).

100% natural light intensity without panicle fertilizer (L1N1), 100% natural light intensity with panicle fertilizer (L1N2), 50% natural light intensity without panicle fertilizer (L2N1) and 50% natural light intensity with panicle fertilizer (L2N2).

Under consistent light conditions, the nitrogen content and accumulation of N2 at maturity stage increased by 17.68% to 21.16% and 32.58% to 37.87%, respectively, compared to N1.

#### 3.2.3 The content of nitrogen in grain

**Figure 6:**
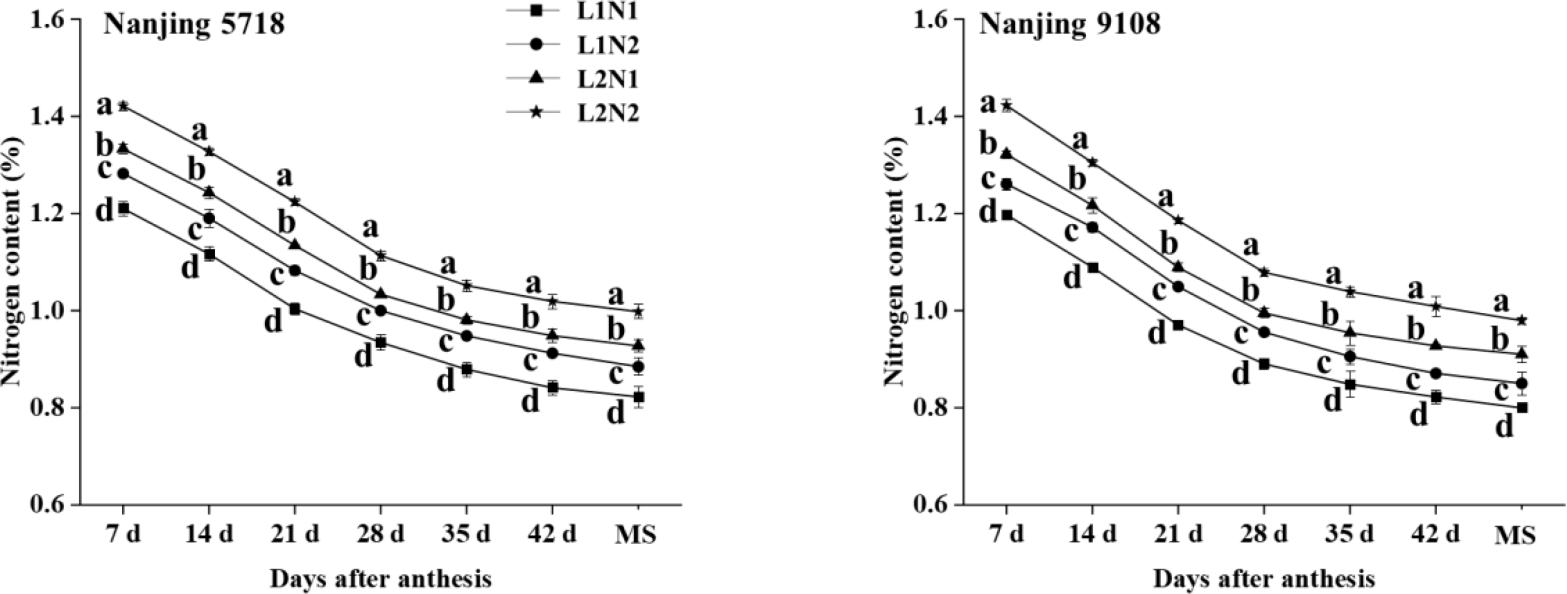
Nitrogen content in the grains of japonica rice after anthesis. Values are mean ± SD (n = 3) and values in the same column with different letters are significantly different (*p* < 0.05). Maturity stage (MS). 100% natural light intensity without panicle fertilizer (L1N1), 100% natural light intensity with panicle fertilizer (L1N2), 50% natural light intensity without panicle fertilizer (L2N1) and 50% natural light intensity with panicle fertilizer (L2N2). .

Under equivalent nitrogen conditions, compared to L1, the nitrogen content of L2 increased by 10.18% to 11.24%, 11.29% to 13.07%, 12.20% to 13.02%, 10.58% to 12.89%, 10.92% to 14.76%, 11.70% to 15.82%, and 12.74% to 15.23%, respectively, from 7 days after anthesis to maturity stage (**Figure 6**).

Under consistent light conditions, compared to N1, the nitrogen content of N2 increased by 5.27% to 6.60%, 6.59% to 8.87%, 7.88% to 8.90%, 7.03% to 8.42%, 6.72% to 8.91%, 5.94% to 8.75%, and 6.27% to 7.64%, respectively, from 7 days after anthesis to maturity stage.

#### 3.2.4 Enzyme activities of nitrogen metabolism in leaves and grains

**Figure 7:**
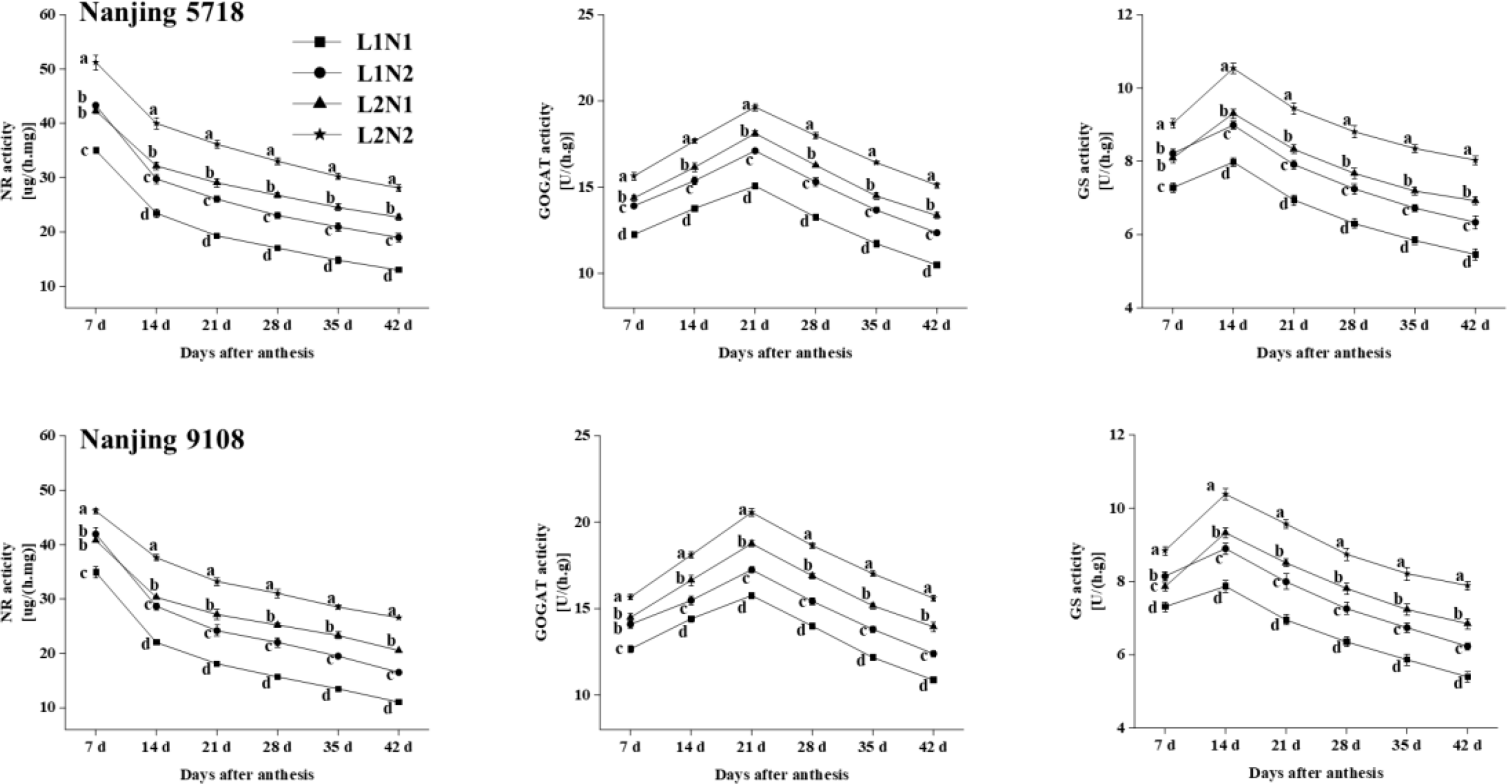
Dynamics of enzyme activities in the leaves and grains of japonica rice. Values are mean ± SD (n = 3) and values in the same column with different letters are significantly different (*p* < 0.05). Nitrate reductase (NR), glutamine synthetase (GS) and glutamate synthase (GOGAT). 100% natural light intensity without panicle fertilizer (L1N1), 100% natural light intensity with panicle fertilizer (L1N2), 50% natural light intensity without panicle fertilizer (L2N1) and 50% natural light intensity with panicle fertilizer (L2N2).

Under equivalent nitrogen conditions, compared to L1, NR in leaves of L2 increased by 10.08% to 85.53% (**Figure 7**), while the activities of GOGAT and GS in grains of L2 increased by 11.15% to 28.36% and 7.54% to 26.86%, respectively, from 7 days to 42 days after anthesis.

Under consistent light conditions, compared to N1, NR in leaves of N2 increased by 13.25% to 49.26%, and the activities of GOGAT and GS in grains of N2 increased by 8.13% to 17.82% and 11.29% to 16.08%, respectively, from 7 days to 42 days after anthesis.

#### 3.2.5 Characterization of protein content and accumulation in brown rice

**Figure 8:**
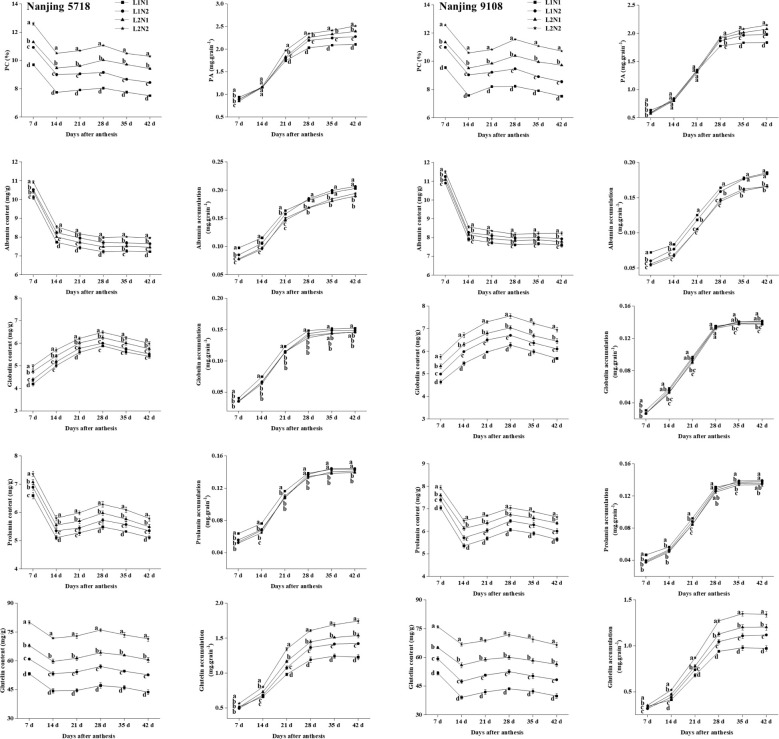
1 Dynamics of protein in the grains of japonica rice. Values are mean ± SD (n = 3) and values in the same column with different letters are significantly different (*p* < 0.05). Protein content (PC) and protein accumulation (PA). 100% natural light intensity without panicle fertilizer (L1N1), 100% natural light intensity with panicle fertilizer (L1N2), 50% natural light intensity without panicle fertilizer (L2N1) and 50% natural light intensity with panicle fertilizer (L2N2). 2 Dynamics of protein in the grains of japonica rice. Values are mean ± SD (n = 3) and values in the same column with different letters are significantly different (*p* < 0.05). Protein content (PC) and protein accumulation (PA). 100% natural light intensity without panicle fertilizer (L1N1), 100% natural light intensity with panicle fertilizer (L1N2), 50% natural light intensity without panicle fertilizer (L2N1) and 50% natural light intensity with panicle fertilizer (L2N2).

Under equivalent nitrogen conditions, compared to L1, the content of total protein, albumin, globulin, prolamin, and glutelin in L2 increased by 11.60% to 29.26%, 1.63% to 4.21%, 6.09% to 15.36%, 6.81% to 14.49%, and 25.76% to 43.01%, respectively, from 7 days to 42 days after anthesis. Furthermore, reflecting the higher MAR of total protein and glutelin in L2 compared to L1 (**Table 4**), the accumulation of total protein and glutelin in L2 increased by 2.84% to 13.62% and 20.00% to 25.30%, respectively, at 42 days after anthesis (**Figure 8**).

**Table 4A:** Characteristics of protein accumulation.

Cultivar	Treatment	Protein			Glutelin		
		MAR (mg grain^-1^ d^-1^)	K	R^2^	MAR (mg grain^-1^ d^-1^)	K	R^2^
Nanjing5718	L1N1	0.0448	2.20	0.94379	0.2619	12.98	0.95280
	L1N2	0.0484	2.37	0.96038	0.3048	14.81	0.96897
	L2N1	0.0507	2.51	0.97149	0.3327	16.12	0.97770
	L2N2	0.0515	2.62	0.96852	0.3634	18.19	0.98111
Nanjing9108	L1N1	0.0387	1.96	0.94698	0.1978	10.37	0.93062
	L1N2	0.0415	2.10	0.96922	0.2331	12.08	0.97090
	L2N1	0.0421	2.21	0.96622	0.2481	12.79	0.97381
	L2N2	0.0427	2.29	0.97385	0.2711	14.15	0.97935

Protein accumulation was modeled using the logarithmic equation y=K/(1+ea−bx). K, the max accumulation. R2, determination coefficient of the equation. Mean accumulation rate (MAR). 100% natural light intensity without panicle fertilizer (L1N1), 100% natural light intensity with panicle fertilizer (L1N2), 50% natural light intensity without panicle fertilizer (L2N1) and 50% natural light intensity with panicle fertilizer (L2N2).

**Table 4B:** Characteristics of protein accumulation.

Cultivar	Treatment	Albumin			Globulin			Prolamin		
		MAR (mg grain^-1^ d^-1^)	K	R^2^	MAR (mg grain^-1^ d^-1^)	K	R^2^	MAR (mg grain^-1^ d^-1^)	K	R^2^
Nanjing5718	L1N1	0.0366	2.14	0.96237	0.0394	1.56	0.98679	0.0297	1.52	0.93488
	L1N2	0.0376	2.17	0.97313	0.0370	1.52	0.99018	0.0288	1.53	0.95202
	L2N1	0.0351	2.01	0.97339	0.0367	1.50	0.99033	0.0296	1.48	0.95276
	L2N2	0.0340	2.08	0.97595	0.0348	1.49	0.99268	0.0288	1.50	0.95696
Nanjing9108	L1N1	0.0314	2.06	0.95945	0.0331	1.45	0.98156	0.0275	1.49	0.93905
	L1N2	0.0324	2.03	0.97930	0.0331	1.46	0.99244	0.0282	1.50	0.96406
	L2N1	0.0291	1.84	0.97025	0.0317	1.43	0.98684	0.0271	1.46	0.96422
	L2N2	0.0277	1.84	0.98126	0.0313	1.44	0.99356	0.0260	1.43	0.97452

Protein accumulation was modeled using the logarithmic equation y=K/(1+ea−bx). K, the max accumulation. R2, determination coefficient of the equation. Mean accumulation rate (MAR). 100% natural light intensity without panicle fertilizer (L1N1), 100% natural light intensity with panicle fertilizer (L1N2), 50% natural light intensity without panicle fertilizer (L2N1) and 50% natural light intensity with panicle fertilizer (L2N2).

Under consistent light conditions, compared to N1, the content of total protein, albumin, globulin, prolamin, and glutelin in N2 increased by 5.37% to 19.35%, 3.26% to 6.90%, 1.76% to 9.58%, 3.25% to 6.68%, and 14.49% to 21.57%, respectively, from 7 days to 42 days after anthesis. Moreover, reflecting the higher MAR of total protein and glutelin in N2 compared to N1, the accumulation of total protein and glutelin in N2 increased by 0.44% to 10.97% and 11.60% to 15.85%, respectively, at 42 days after anthesis.

### 3.3 Differences in C/N of grains under different light and nitrogen fertilizer conditions

Under equivalent nitrogen conditions, compared to L1, C/N of L2 was decreased by 25.86% to 33.19%, 24.76% to 32.81%, 26.81% to 31.95%, 27.34% to 30.47%, 27.05% to 32.79%, 27.69% to 33.95%, and 30.45% to 37.10%, respectively, from 7 days after anthesis to maturity stage (**Table 5**).

**Table 5:** Dynamics of C/N in the grains of japonica rice after anthesis.

Cultivar	Treatment	7d	14d	21d	28d	35d	42d	MS
Nanjing5718	L1N1	21.45 ± 0.08a	36.12 ± 0.09a	53.07 ± 0.86a	60.26 ± 1.34a	61.51 ± 0.87a	61.30 ± 3.22a	61.21 ± 0.88a
	L1N2	18.49 ± 0.52c	31.90 ± 0.51c	46.53 ± 0.98c	53.37 ± 0.44b	54.05 ± 0.80b	53.66 ± 1.58c	54.24 ± 2.86b
	L2N1	15.16 ± 0.26d	26.86 ± 0.41d	37.58 ± 0.83d	43.79 ± 0.96c	43.92 ± 1.16c	42.95 ± 1.30e	42.16 ± 1.44d
	L2N2	12.46 ± 0.21e	22.84 ± 0.14e	31.66 ± 0.58e	37.11 ± 0.23d	36.65 ± 0.11d	35.45 ± 1.18f	34.11 ± 0.33e
Nanjing9108	L1N1	20.19 ± 0.12b	35.63 ± 0.40b	51.89 ± 1.66b	61.60 ± 0.07a	61.54 ± 1.00a	58.98 ± 1.66b	59.50 ± 1.07a
	L1N2	17.86 ± 0.79c	31.48 ± 0.75c	45.54 ± 0.66c	54.30 ± 0.51b	53.79 ± 0.59b	51.64 ± 1.14d	52.11 ± 2.20c
	L2N1	14.97 ± 0.24d	26.81 ± 0.90d	37.97 ± 0.95d	44.73 ± 0.83c	44.89 ± 0.43c	42.64 ± 0.98e	41.38 ± 1.13d
	L2N2	11.93 ± 0.25e	21.15 ± 0.87e	31.47 ± 0.65e	37.91 ± 0.74d	36.15 ± 0.52d	34.33 ± 0.47f	33.57 ± 0.84e

Values are mean ± SD (n = 3) and values in the same column with different letters are significantly different (*p* < 0.05). Maturity stage (MS).

100% natural light intensity without panicle fertilizer (L1N1), 100% natural light intensity with panicle fertilizer (L1N2), 50% natural light intensity without panicle fertilizer (L2N1) and 50% natural light intensity with panicle fertilizer (L2N2).

Under consistent light conditions, compared to N1, C/N of N2 was decreased by 11.55% to 20.29%, 11.65% to 21.10%, 12.21% to 17.12%, 11.44% to 15.25%, 12.12% to 19.46%, 12.43% to 19.51%, and 11.40% to 19.07%, respectively, from 7 days after anthesis to maturity stage.

### 3.4 Differences in carbon and nitrogen metabolism products and eating quality at maturity stage under different light and nitrogen fertilizer conditions

#### 3.4.1 The starch, protein and its components of japonica rice (milled rice)

We further investigated the starch, protein, and their components in milled rice, which is commonly used for cooking purposes. The data concerning starch and protein in milled rice under various treatments exhibited a consistent trend with the related data observed in brown rice (**Figure 4**; **Figure 8**). Under equivalent nitrogen conditions, compared to L1, the TSC, AC, and APC of L2 decreased by 4.30% to 5.59%, 6.87% to 11.52%, and 3.62% to 5.42%, respectively (**Table 6**). Moreover, when compared to L1, the content of total protein, albumin, globulin, prolamin, and glutelin in L2 increased by 21.31% to 29.70%, 3.74% to 15.36%, 9.98% to 20.88%, 9.61% to 15.14%, and 22.81% to 49.28%, respectively (**Table 7**).

**Table 6:** Starch and components in milled rice of japonica rice.

Cultivar	Treatment	TSC(%)	AC(%)	APC(%)
Nanjing5718	L1N1	76.67 ± 0.41a	9.61 ± 0.07a	67.06 ± 0.35a
	L1N2	75.44 ± 0.40ab	9.15 ± 0.09b	66.29 ± 0.49a
	L2N1	73.37 ± 0.94cd	8.95 ± 0.04c	64.42 ± 0.89bc
	L2N2	71.22 ± 0.09ef	8.52 ± 0.06d	62.70 ± 0.09d
Nanjing9108	L1N1	75.69 ± 0.74ab	9.15 ± 0.10b	66.54 ± 0.66a
	L1N2	74.31 ± 0.38bc	8.68 ± 0.05d	65.63 ± 0.42ab
	L2N1	72.26 ± 0.27de	8.13 ± 0.05e	64.13 ± 0.24c
	L2N2	70.21 ± 0.91f	7.68 ± 0.03f	62.53 ± 0.89d

Values are mean ± SD (n = 3) and values in the same column with different letters are significantly different (*p* < 0.05). Total starch content (TSC), amylose content (AC) and amylopectin content (APC).

100% natural light intensity without panicle fertilizer (L1N1), 100% natural light intensity with panicle fertilizer (L1N2), 50% natural light intensity without panicle fertilizer (L2N1) and 50% natural light intensity with panicle fertilizer (L2N2).

**Table 7:** Protein and components in milled rice of japonica rice.

Cultivar	Treatment	PC (%)	Content (mg/g)			
			Albumin	Globulin	Prolamin	Glutelin
Nanjing5718	L1N1	6.66 ± 0.09f	2.56 ± 0.05f	3.97 ± 0.03de	5.37 ± 0.02d	45.19 ± 0.42d
	L1N2	8.16 ± 0.07e	2.74 ± 0.03e	4.03 ± 0.05cd	5.64 ± 0.08cd	58.89 ± 2.34c
	L2N1	8.35 ± 0.07d	2.70 ± 0.06e	4.37 ± 0.01b	5.88 ± 0.14bc	60.42 ± 0.95c
	L2N2	10.03 ± 0.13a	3.16 ± 0.06d	4.54 ± 0.08b	6.38 ± 0.04a	76.74 ± 0.69a
Nanjing9108	L1N1	6.68 ± 0.08f	3.23 ± 0.01cd	3.72 ± 0.06e	5.41 ± 0.07d	41.00 ± 0.12d
	L1N2	8.11 ± 0.07e	3.46 ± 0.04b	4.28 ± 0.07bc	5.83 ± 0.04c	57.39 ± 1.41c
	L2N1	8.67 ± 0.07c	3.35 ± 0.01bc	4.50 ± 0.06b	6.23 ± 0.04ab	61.20 ± 1.65bc
	L2N2	9.84 ± 0.12b	3.60 ± 0.02a	5.11 ± 0.05a	6.41 ± 0.01a	70.49 ± 1.69ab

Values are mean ± SD (n = 3) and values in the same column with different letters are significantly different (*p* < 0.05). Protein content (PC).

100% natural light intensity without panicle fertilizer (L1N1), 100% natural light intensity with panicle fertilizer (L1N2), 50% natural light intensity without panicle fertilizer (L2N1) and 50% natural light intensity with panicle fertilizer (L2N2).

Under consistent light conditions, compared to N1, the TSC, AC and APC of N2 decreased by 1.60% to 2.93%, 4.79% to 5.54% and 1.15% to 2.67%, respectively. Additionally, when compared to N1, the content of total protein, albumin, globulin, prolamin and glutelin in N2 increased by 13.53% to 22.47%, 7.17% to 17.07%, 1.44% to 15.09%, 2.84% to 8.42% and 15.17% to 40.00%, respectively.

#### 3.4.2 The eating quality of japonica rice (milled rice)

Under equivalent nitrogen conditions, compared to L1, the appearance, adhesiveness, elasticity, and taste values of cooked L2 rice decreased by 13.24% to 14.77%, 7.46% to 8.33%, 1.10% to 3.96% and 10.06% to 11.42%, respectively, while the hardness of cooked L2 rice increased by 13.17% to 13.99% (**Table 8**).

**Table 8:** The eating quality of rice.

Cultivar	Treatment	Texture Properties			Taste Analyzer Properties	
		Hardness (g)	Elasticity (%)	Adhesiveness (g)	Appearance	Taste Value
Nanjing5718	L1N1	116.83 ± 1.34d	0.496 ± 0.002b	1373.07 ± 10.88a	8.90 ± 0.15a	86.25 ± 1.10a
	L1N2	123.95 ± 2.13c	0.505 ± 0.002a	1317.78 ± 8.74b	8.10 ± 0.05b	80.46 ± 0.10b
	L2N1	133.18 ± 2.60b	0.490 ± 0.003c	1260.92 ± 20.61d	7.66 ± 0.11c	76.59 ± 0.79c
	L2N2	140.72 ± 1.32a	0.485 ± 0.002d	1219.42 ± 7.49e	6.99 ± 0.20d	72.16 ± 1.16d
Nanjing9108	L1N1	118.87 ± 2.25d	0.499 ± 0.002b	1354.20 ± 11.73a	8.80 ± 0.17a	85.55 ± 1.35a
	L1N2	124.56 ± 1.49c	0.502 ± 0.002a	1292.50 ± 22.02c	8.16 ± 0.06b	80.78 ± 0.47b
	L2N1	135.33 ± 1.07b	0.489 ± 0.002c	1241.40 ± 4.25de	7.50 ± 0.11c	75.78 ± 1.04c
	L2N2	140.96 ± 1.13a	0.487 ± 0.002cd	1192.45 ± 12.99f	7.08 ± 0.05d	72.65 ± 0.80d

Values are mean ± SD (n = 3) and values in the same column with different letters are significantly different (*p* < 0.05).

100% natural light intensity without panicle fertilizer (L1N1), 100% natural light intensity with panicle fertilizer (L1N2), 50% natural light intensity without panicle fertilizer (L2N1) and 50% natural light intensity with panicle fertilizer (L2N2).

Under consistent light conditions, compared to N1, the appearance, adhesiveness, and taste values of cooked N2 rice decreased by 5.60% to 8.99%, 3.29% to 4.56% and 4.13% to 6.71%, respectively, while hardness of cooked N2 rice increased by 4.18% to 6.09%.

### 3.5 Relationships between C/N and carbon and nitrogen metabolism and rice eating quality

#### 3.5.1 Correlation analysis between carbon and nitrogen metabolism-related enzymes and products and C/N

**Figure 9:**
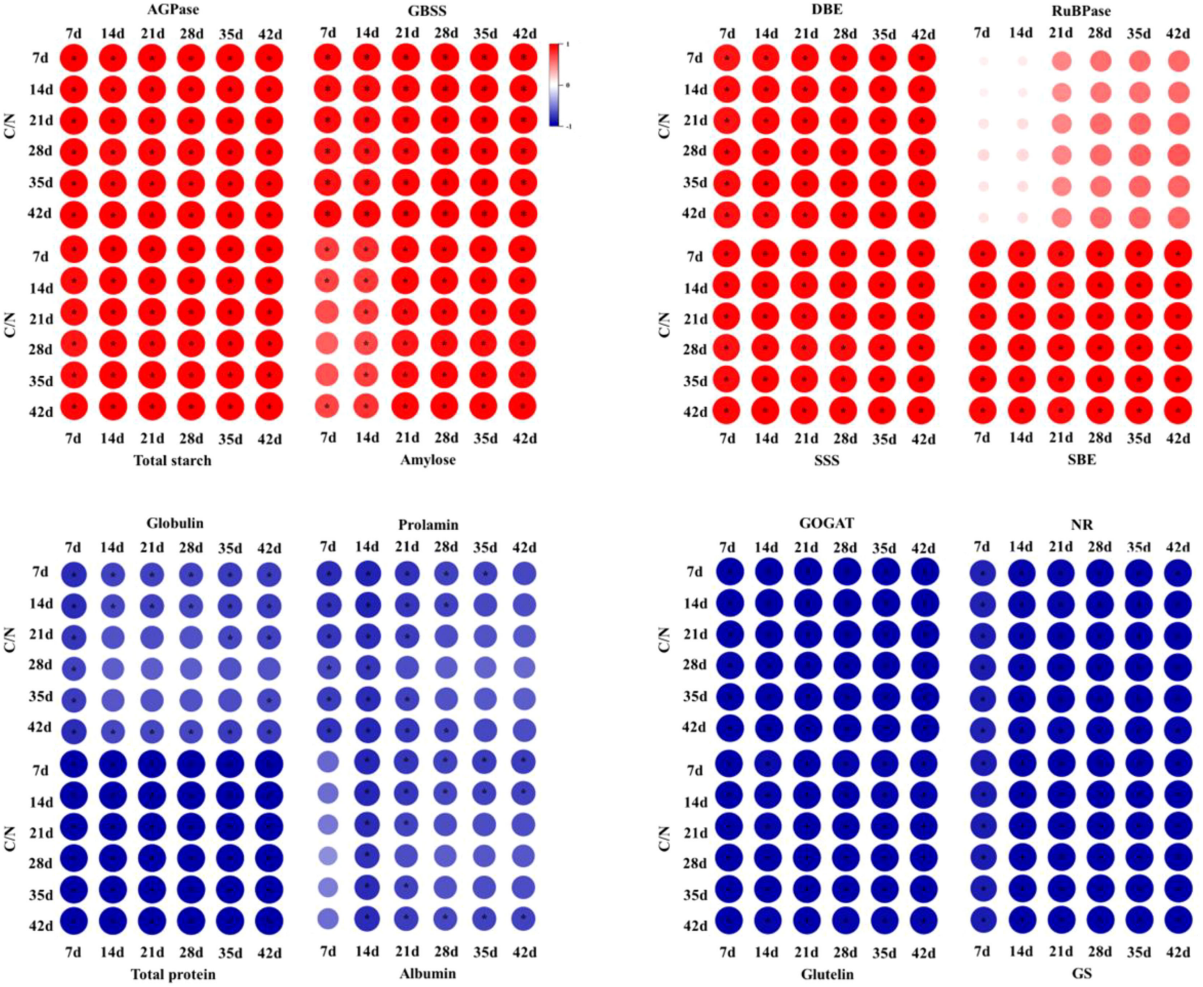
Correlation analysis between carbon and nitrogen metabolism-related enzymes and products and C/N. The carbon and nitrogen metabolites depicted in the figure refer to their respective contents. 1,5-bisphosphate carboxylase (RuBPase), ADP-glucose pyrophosphorylase (AGPase), granule-bound starch synthase (GBSS), soluble starch synthase (SSS), starch branching enzyme (SBE), starch debranching enzyme (DBE), nitrate reductase (NR), glutamine synthetase (GS) and glutamate synthase (GOGAT). Red represents positive correlation and blue represents negative correlation. The size of the circles, the intensity of their colors and * represents the degree of correlation.

The correlation analysis revealed a strong association between carbon and nitrogen metabolism and C/N (**Figure 9**). Regarding metabolic enzymes, the activities of enzymes involved in carbon metabolism displayed a positive correlation with C/N, while the activity of enzymes related to nitrogen metabolism exhibited a negative correlation with C/N. Notably, enzymes such as AGPase, GBSS, SSS, SBE, and DBE in grains demonstrated significant or highly significant positive correlations with C/N across the entire grain filling period. In contrast, NR in leaves, and GS and GOGST in grains displayed significant or highly significant negative correlations with the C/N across the entire grain filling period. Concerning metabolic products, starch and amylose, as carbon metabolic products, exhibited significant or highly significant positive correlations with C/N throughout the entire grain filling stage. Conversely, nitrogen metabolic products, particularly proteins, notably glutelin, demonstrated significant or highly significant negative correlations with C/N during the grain filling stage. Additionally, albumin, globulin, and prolamin exhibited negative or significantly negative correlations with C/N during the grain filling stage.

#### 3.5.2 Correlation analysis between C/N and rice eating quality

**Figure 10:**
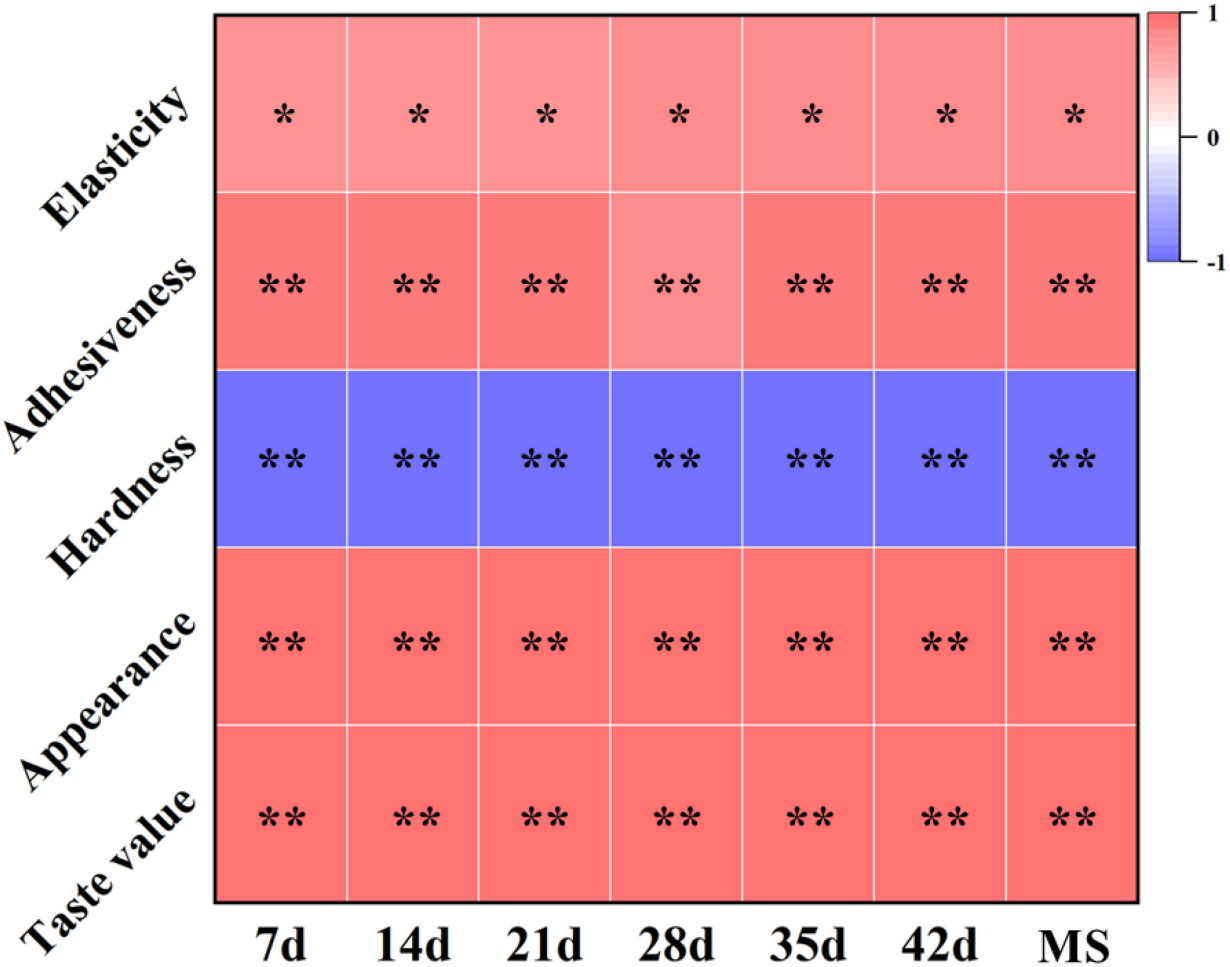
Correlation analysis between C/N in grains after anthesis and rice eating quality. Red represents positive correlation and blue represents negative correlation. The intensity of colors, * and **represents the degree of correlation. * Indicates significant correlation (P<0.05) and ** indicates highly significant correlation (P<0.01). Maturity stage (MS).

In this study, a strong correlation was observed between the C/N in grains and both carbon and nitrogen metabolism (**Figure 9**). Higher C/N ratios in grains were associated with robust carbon metabolic capacity but weaker nitrogen metabolic capacity. As the C/N ratio decreased, carbon metabolism gradually weakened while nitrogen metabolism gradually enhanced (**Figure 9**). Subsequently, we performed a correlation analysis between the C/N ratio and rice eating quality to investigate the relationship between carbon and nitrogen metabolism and rice eating quality (**Figure 10**). The findings revealed a significant or highly significant positive correlation between the C/N and attributes such as elasticity, adhesiveness, appearance, and taste value of rice. Conversely, a significant or highly significant negative correlation was observed between the C/N ratio and hardness. These results suggest that the level of carbon metabolism exhibited a positive association with rice eating quality, while nitrogen metabolism showed a negative correlation with rice eating quality.

## 4 Discussion

### 4.1 Differences in carbon and nitrogen metabolism of japonica rice during filling stage under different light and nitrogen fertilizer conditions

In this study, compared with L1, the activity of RuBPase in L2 leaves was reduced (**Figure 3**), leading to a decreased capacity to assimilate CO_2_ (Parry et al., 2012). Consequently, the net photosynthetic rate in L2 leaves declined (**Figure 1**). This reduction in photosynthetic efficiency resulted in inadequate carbohydrate accumulation, thereby contributing to a decreased NSC content in both the stems and grains of L2 (**Table 1**; **Figure 2**). Additionally, we observed reduced activities of starch synthesis enzymes, including AGPase, GBSS, SSS, SBE, and DBE in L2 grains (**Figure 3**), which constrained the synthesis of both amylose and amylopectin (Jeng et al., 2007; Pfister and Zeeman, 2016; Wang et al., 2021). Consequently, the MAR, content, and accumulation of starch in L2 grains were all reduced compared to those in L1 (**Table 2**; **Figure 4**). Carbon and nitrogen metabolism are intricately interconnected, with nitrogen metabolism depending on carbon metabolism to supply the essential carbon skeletons (Nunes-Nesi et al., 2010; Xu et al., 2023b). The results of this study reveal that the carbon metabolic capacity of L2 was inferior to that of L1. This suggests that the carbon metabolism of L2 was insufficient in supplying an adequate amount of carbon skeletons for nitrogen assimilation and translocation, consequently impeding the normal transference of nitrogen from leaves to stems and grains. As a consequence, the leaves of L2 sustained an extended green period, as indicated by elevated SPAD values (**Figure 5**). Nonetheless, under low light conditions, the presence of blue and violet light enhances nitrogen synthesis (Chen et al., 2019; Wang et al., 2015). An increased nitrogen supply compensated for the deficiency in nitrogen translocation from the leaves, resulting in higher nitrogen levels in both the stems and grains of L2 (**Table 3**; **Figure 6**). Additionally, our observations indicated elevated activities of GS and GOGAT in L2 grains (**Figure 7**), known to enhance protein synthesis (Fei et al., 2023; Tang et al., 2018). Consequently, the protein content of L2, comprising albumin, globulin, prolamin, and glutelin, exceeded that of L1 (**Table 4**; **Figure 8**). In conclusion, a 50% reduction in light intensity inhibited carbon metabolism but promoted nitrogen metabolism, resulting in a decrease in the carbon-to-nitrogen ratio in grains. This transition ultimately led to a decrease in starch content and an increase in protein content in the endosperm.

Nitrogen fertilization is a crucial factor in influencing nitrogen metabolism. Previous studies have found that increased nitrogen fertilization can slow down chlorophyll breakdown, thereby postponing leaf senescence (Chen et al., 2023). Consistent with these findings, our study observed a comparable phenomenon wherein leaves exhibited sustained high SPAD values following the application of panicle fertilizer (N2) (**Figure 5**). NR serves as a pivotal enzyme in nitrogen assimilation, and its heightened activity facilitates nitrate reduction, thereby accelerating nitrogen assimilation (Liu et al., 2022). In this study, the NR activity in N2 leaves was significantly higher than in N1 (**Figure 7**), facilitating nitrogen assimilation and accumulation. Additionally, studies have demonstrated that the application of nitrogen fertilizer during the later stages of growth can enhance root development and maintain optimal root activity, leading to increased nitrogen absorption and utilization by the roots (Chen et al., 2023; Xu et al., 2023a). Consequently, the nitrogen content in the stems and grains of N2 surpassed that of N1 (**Table 3**; **Figure 6**). Furthermore, elevated activities of GOGAT and GS were observed in N2 grains (**Figure 7**). The ample nitrogen availability and heightened enzyme activities supported protein synthesis and accumulation (**Table 4**), leading to elevated levels of albumin, globulin, prolamin, and glutelin in the endosperm of N2 in comparison to N1 (**Figure 8**) (Fei et al., 2023; Tang et al., 2018). Nitrogen fertilizer, besides influencing nitrogen metabolism, also impacts carbon metabolism. In this study, compared to N1, the activity of RuBPase in N2 leaves was increased (**Figure 3**), resulting in an enhanced photosynthetic rate of the leaves (**Figure 1**), promoting the synthesis of more carbohydrates (Parry et al., 2012). However, we observed that the NSC content in the stems and grains of N2 was lower than that of N1 (**Table 1**; **Figure 2**). This observation may be attributed to the significant decrease in SBE activity and its mRNA expression in rice plants under high nitrogen fertilizer conditions, consequently inhibiting starch synthesis (Hu, 2020; Hirano et al., 2005). Moreover, compared to N1, the activities of enzymes associated with starch synthesis in N2 grains were reduced (**Figure 3**), consequently impeding the synthesis of both amylose and amylopectin (**Table 2**) (Fei et al., 2023; Tang et al., 2018). Consequently, the TSC, AC, and APC of N2 were lower than those of N1 (**Figure 4**). In conclusion, the application of panicle fertilizer suppressed carbon metabolism while enhancing nitrogen metabolism, resulting in a reduction in the carbon-to-nitrogen ratio in grains, an elevation in protein content, and a reduction in starch content in the endosperm.

### 4.2 Relationship between carbon and nitrogen metabolism and rice eating quality

The present study unveiled notable differences in carbon and nitrogen metabolism of the same japonica rice cultivar across varying light and nitrogen fertilizer treatments, highlighting their impact on the ultimate rice eating quality. Previous research has indicated that the metabolic capacity of rice in terms of carbon and nitrogen can be assessed, to a certain degree, by the C/N ratio (Sun et al., 2020, 2017). In our investigation, the C/N ratio in grains under various light and nitrogen conditions exhibited the following order: L1N1 > L1N2 > L2N1 > L2N2. Subsequent analysis indicated that higher C/N ratios in grains were associated with enhanced carbon metabolism and relatively reduced nitrogen metabolism (**Figure 9**), a trend that aligns with previous observations (Chen et al., 2023). To investigate the relationship between carbon and nitrogen metabolism and rice eating quality, a correlation analysis was conducted between the C/N ratio and taste value. The results revealed a positive correlation between the C/N ratio and rice eating quality (**Figure 10**), indicating that elevated carbon metabolism levels are associated with improved rice eating quality, while nitrogen metabolism exhibits a contrasting influence. In our study, elevated levels of carbon metabolism were associated with abundant NSC in grains, accompanied by increased enzyme activities of APGase, GBSS, SSS, SBE, and DBE, which facilitated starch synthesis and consequently resulted in an increase in amylose content (**Figure 9**). Nevertheless, it is commonly understood that a high AC is generally detrimental to the development of rice eating quality (Li et al., 2023; Tao et al., 2019), which appears to contradict our observation of a positive correlation between carbon metabolism levels and rice eating quality (**Figure 10**). In reality, there exists a trade-off relationship between carbon and nitrogen metabolism (Nunes-Nesi et al., 2010; Sun et al., 2013). Elevated carbon metabolism is frequently associated with reduced nitrogen metabolism, which can slow down nitrogen assimilation in rice plants, resulting in diminished nitrogen content and inadequate activities of GS and GOGAT in the grain. Consequently, this scenario leads to sluggish protein synthesis and a notable decrease in protein content (**Figure 9**). The reduced protein content counteracts the adverse effects of high AC on the texture of cooked rice (Saleh, 2017; Shi et al., 2023), thereby improving rice eating quality with increasing levels of carbon metabolism. Nitrogen metabolism levels exhibited a negative correlation with rice eating quality (**Figure 10**). Our study revealed that higher nitrogen metabolism levels were associated with grains containing abundant nitrogen content and increased activities of GS and GOGAT enzymes. These nitrogen and enzymes accelerate protein synthesis, leading to a significant rise in protein content, particularly the glutelin. Intensified nitrogen metabolism implies a decline in carbon metabolism, impeding starch synthesis and decreasing amylose content (**Figure 9**). Despite the favorable impact of low amylose content on rice eating quality, it is insufficient to counterbalance the adverse effects of high protein content on the texture of cooked rice (Yin et al., 2023; Zhang et al., 2021b). Hence, heightened nitrogen metabolism results in inferior rice eating quality. In conclusion, carbon and nitrogen metabolism are intricately linked to rice eating quality, collectively influencing the synthesis of starch and protein in the endosperm through the regulation of carbon and nitrogen substrates and enzymes, thereby affecting rice eating quality.

## 5 Conclusion

In this study, a 50% reduction in light intensity suppressed carbon metabolism while enhancing nitrogen metabolism, leading to a reduction in the C/N ratio, decreased starch content, increased PC, and consequently, inferior rice eating quality. Conversely, the use of panicle fertilizer enhanced nitrogen metabolism while limiting carbon metabolism, resulting in a similar outcome of reduced C/N ratio, elevated PC, and diminished starch content, contributing to suboptimal rice eating quality. The C/N ratio could serve as an indicator of the carbon and nitrogen metabolic capacities, where a higher ratio indicates strong carbon metabolism and comparatively weaker nitrogen metabolism. Carbon and nitrogen metabolism were closely linked to rice eating quality, with heightened carbon metabolism favoring superior rice eating quality, while nitrogen metabolism had a contrasting impact. In summary, notable variances in carbon and nitrogen metabolism were observed within the same japonica rice cultivar under diverse light and nitrogen fertilizer conditions. These metabolic differences impact the synthesis of starch and protein in the endosperm, ultimately influencing the quality of rice. Our findings suggest that the regulation of carbon and nitrogen metabolism in rice through breeding or cultivation measures in the future represents a pivotal and promising approach to improving the rice eating quality.

## Data Availability

The original contributions presented in the study are included in the article/supplementary material. Further inquiries can be directed to the corresponding author.
